# Acute Gallstone Pancreatitis in Pregnancy: A Multidisciplinary Approach

**DOI:** 10.7759/cureus.50945

**Published:** 2023-12-22

**Authors:** Mark Ayoub, Muhammed Ceesay, Carol Faris, Michael Iannetti

**Affiliations:** 1 Internal Medicine, West Virginia University School of Medicine, Charleston, USA; 2 Internal Medicine, Charleston Area Medical Center, Charleston, USA; 3 General Surgery, Marshall University Joan C. Edwards School of Medicine, Huntington, USA

**Keywords:** surgery general, gastroenterology and endoscopy, obstetric, multidisciplinary decision-making, induced labor, laporoscopic cholecystectomy, three trimesters of pregnancy, gallstone pancreatitis

## Abstract

A common cause of gastrointestinal-related hospitalizations in the United States of America is acute pancreatitis (AP), with an annual incidence of up to 80 cases per 100,000 people. The incidence of AP in pregnancy varies and is approximately 1 in 1000 to 1 in 10,000 births due to the prevalence of obesity and gallstone-related conditions. Deciding on the timing of surgical intervention in acute biliary pancreatitis during pregnancy remains challenging, and there are no consensus recommendations. Gallstone pancreatitis has a high recurrence rate of up to 50% during the first trimester. A 30-year-old G3P2 at 34 weeks of gestation presented to the emergency room (ER) with recurrent intermittent sudden severe epigastric and right upper quadrant abdominal pain radiating to the back. She had no history of alcohol consumption, and lipid studies were normal on presentation. A right upper quadrant ultrasound scan showed cholelithiasis without signs of acute cholecystitis and a common bile duct diameter of 0.5 cm. However, her serum lipase level was 824, compared to normal levels on her previous ER visits. Other significant labs included elevated alkaline phosphatase (ALP) of 125 and mild transaminitis, with alanine transaminase (ALT) and aspartate aminotransferase (AST) levels of 84 and 57, respectively. She was admitted on account of suspected gallstone pancreatitis and was treated supportively with IV fluids and adequate pain control with opioids. A subsequent magnetic resonance cholangiopancreatography (MRCP) revealed no obvious choledocholithiasis. After consultation with the obstetrics, gastroenterology, and general surgery teams, it was decided to defer cholecystectomy until after delivery. The patient was induced at 36 weeks of gestation, and she had an uneventful vaginal delivery. Two weeks later, she had an elective laparoscopic cholecystectomy with no complications.

## Introduction

A common cause of gastrointestinal-related hospitalizations in the United States of America is acute pancreatitis (AP), with an annual incidence of up to 80 cases per 100,000 people [1]. The incidence of AP in pregnancy varies and is approximately 1 in 1000 to 1 in 10,000 births due to the prevalence of obesity and gallstone-related conditions. Deciding on the timing of surgical intervention in acute biliary pancreatitis during pregnancy remains challenging, and there are no consensus recommendations [2]. Indications for surgery include but are not limited to severe uncontrolled symptoms, obstructive jaundice, acute cholecystitis, resistance to medical treatment, peritonitis, or hemodynamic instability. With recent surgical improvements, it has been shown that laparoscopic surgery is safe in all the trimesters of pregnancy. Notably, the risk of recurrence of gallstone pancreatitis in pregnancy is high, with one study reporting a recurrence rate of up to 50% in pregnant females during the first trimester [3]. 

## Case presentation

A 30-year-old gravida 3 para 2 at 34 weeks of gestation who is without significant past medical history presented to the emergency room (ER) with severe abdominal pain. The pain was sudden in onset, intermittent in nature, mainly in the epigastrium and right upper quadrant (RUQ), and radiates to the back. She presented to the ER multiple times with a similar presentation since the 24th week of pregnancy. She did not have any alcohol use history and did not take any medications regularly. She did not have any history of similar pain in her previous pregnancies.

On physical exam, she was tachycardic with a heart rate in the 100s and normotensive. She was slightly diaphoretic; however, her temperature was normal. There were no scleral icterus, palmar erythema, or jaundice. There was no lymphadenopathy. She had significant epigastric and RUQ tenderness to deep palpation with a positive Murphy’s sign. Otherwise, the abdomen was soft, with no rebound or guarding.

Her initial lab work evaluation is shown in Table 1, which showed mild transaminitis; alanine aminotransferase (ALT) was 84 U/L, and aspartate aminotransferase (AST) was 57 U/L. She had a normal white cell count, kidney function, and electrolytes. Her serum lipase was significantly elevated at 824 U/L. Her total bilirubin was slightly elevated at 1.8 mg/dl with a direct component of 1.11 mg/dl. 

**Table 1:** Relevant inpatient lab values

Labs	Day 1 (normal range)	Day 2
Serum lipase	824 U/L (11-82 U/L)	96 U/L
Alkaline phosphatase	144 U/L (34-104 U/L)	125 U/L
Alanine transaminase	126 U/L (7-52 U/L)	84 U/L
Aspartate transaminase	109 U/L (13-39 U/L)	57 U/L
Total bilirubin	1.8 mg/dL (0.3-1.0 mg/dL)	0.6 mg/dL
Direct bilirubin	1.11 mg/dl (0.03-0.18 mg/dL)	-

**Figure 1:**
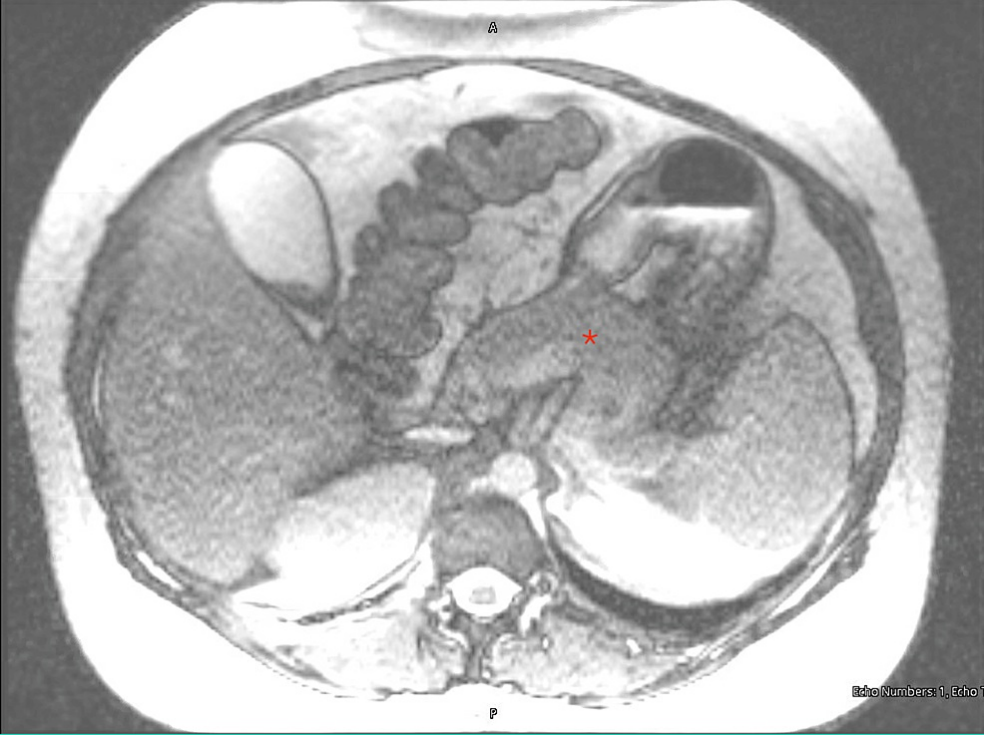
Magnetic resonance cholangiopancreatography with a red star showing a diffusely swollen pancreas

**Figure 2:**
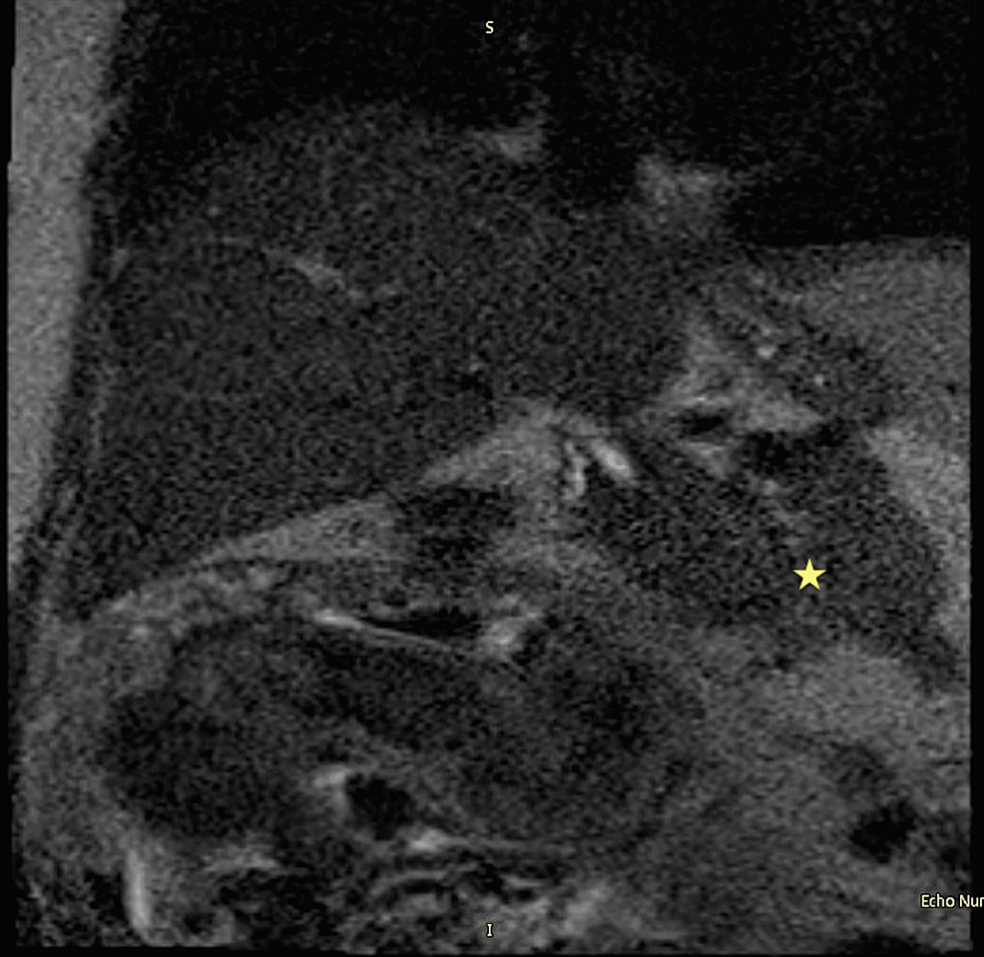
Magnetic resonance cholangiopancreatography with a yellow star showing a diffusely swollen pancreas

**Figure 3:**
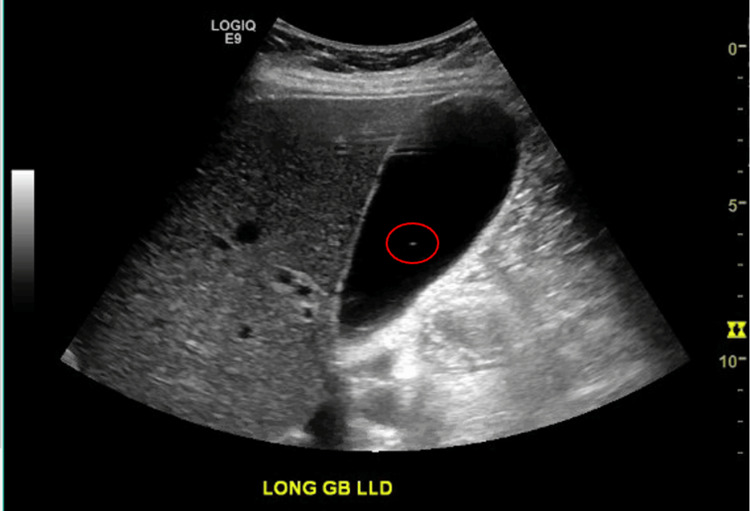
Ultrasound with a red circle showing non-obstructing gallstone

**Figure 4:**
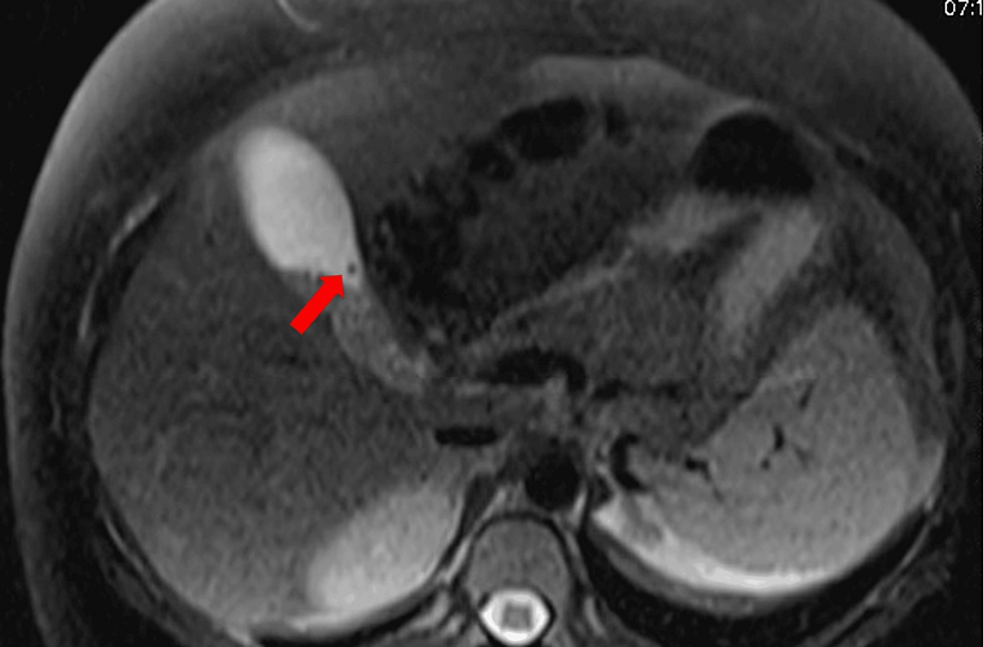
Magnetic resonance cholangiopancreatography with a red arrow showing non-obstructing gallstone

**Figure 5:**
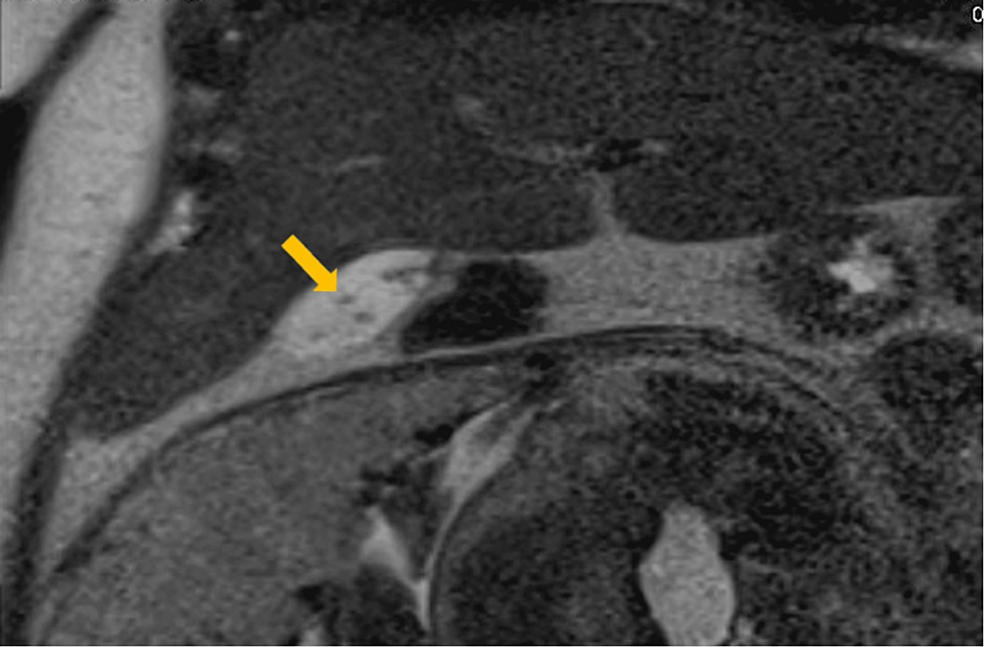
Magnetic resonance cholangiopancreatography with a yellow arrow showing multiple small gallstones

A RUQ ultrasound (US) was performed and revealed cholelithiasis without signs of acute cholecystitis (Figure 1). The common bile duct (CBD) diameter was normal at 0.5cm. She was admitted with gallstone pancreatitis and was treated supportively with intravenous (IV) fluids and pain control. A magnetic resonance cholangiopancreatography (MRCP) was performed and confirmed the diagnosis of mild acute pancreatitis (Figures 2, 3) and revealed numerous gallstones (Figures 4, 5) but with no choledocholithiasis.

Repeat lab work on day two showed down trending transaminitis with a lipase of 96 U/L. Her pain was controlled with opiates.

Surgery, gastroenterology, and obstetric services were consulted, and after extensive discussions with the patient, her family, and the consultants, cholecystectomy was deferred until after delivery. She was induced at 36 weeks of gestation and had an uneventful vaginal delivery. Two weeks later, she underwent elective laparoscopic cholecystectomy with no complications.

## Discussion

Acute pancreatitis in pregnancy is not uncommon. The incidence in the general population is 6 in 10.000, and in pregnant females, it is up to 1 in 10.000 [4-6]. The most common etiologies are the same as in the general population, which include gallstones, alcohol, and hypertriglyceridemia, with gallstones causing up to 70% of the cases [7, 8]. The diagnostic criteria are also the same as in the general population: characteristic abdominal pain, evidence of pancreatic inflammation on imaging, and elevation of lipase/amylase more than three times the upper normal limit. Imaging modality may be challenging in pregnancy due to the risk of radiation to the fetus. US is a great initial modality and can identify gallstones and evaluate the CBD. CT has a higher risk of radiation; therefore, we tend to use MRCP, which is 92% sensitive and does not have significant radiation exposure for both the mom and the fetus [9]. Endoscopic retrograde cholangiopancreatography (ERCP) is safe in pregnancy and is usually reserved for severe biliary pancreatitis with any evidence of cholangitis, CBD obstruction, or lack of clinical improvement [10]. In pregnant females with gallstone pancreatitis, two major decisions need to be made: first is the choice of procedure to clear the biliary obstruction, and second is the timing of the procedure [2]. Factors that affect the decision include pregnancy trimester, CBD dilation, presence of cholangitis or sepsis, and severity of pancreatitis. Early cholecystectomy is therapeutic and prophylactic to prevent recurrence of pancreatitis [11-13]. Cholecystectomy is safe in any trimester, being safest in the second trimester [14, 15]. When performed in the third-trimester preterm labor may develop; however, that is usually successfully treated with tocolytics without harm to either the mom or the fetus [16]. Making those major decisions needs extensive multidisciplinary discussions with general surgery, gastroenterology, obstetric services, the patient, and their family using the above-mentioned evidence to make the safest decision and ensure the best outcome for both the mother and the fetus.

## Conclusions

In pregnant patients, the management of gallstone pancreatitis requires multidisciplinary discussions. Management should be done on a case-by-case basis, considering patient factors. The most common cause of AP in pregnancy is gallstones. Diagnostic modalities such as abdominal ultrasound, CT scan, endoscopic ultrasound (EUS), and magnetic resonance cholangiopancreatography (MRCP) are used to identify the biliary cause/obstruction causing AP. The use of CT scans is limited due to the potential risk of fetal radiation. It is advised to avoid diagnostic endoscopic retrograde cholangiopancreatography (ERCP) whenever possible, as it is invasive with a risk of complications, including bleeding, perforation, pancreatitis, and fetal radiation. On the other hand, non-contrasted studies such as RUQ ultrasound, MRCP, and EUS do not pose these risks.

The general approach to managing AP during pregnancy involves supportive measures such as intravenous fluids, pain control, and early feeding. Ideally, laparoscopic cholecystectomy is performed during the second trimester while the risk to the fetus is minimal, and surgical technical challenges are limited as the uterus is still enlarging without significant mass effect. We wanted to present this case to highlight how the management of acute gallstone pancreatitis during pregnancy can differ from one patient to the other depending on their clinical condition, advancement of pregnancy, and multidisciplinary discussions.
